# *massPix*: an R package for annotation and interpretation of mass spectrometry imaging data for lipidomics

**DOI:** 10.1007/s11306-017-1252-5

**Published:** 2017-09-21

**Authors:** Nicholas J. Bond, Albert Koulman, Julian L. Griffin, Zoe Hall

**Affiliations:** 1 MRC Human Nutrition Research, 120 Fulbourn Road, Cambridge, CB1 9NL UK; 2 Department of Biochemistry and Cambridge Systems Biology Centre, University of Cambridge, 80 Tennis Court Road, Cambridge, CB2 1GA UK

**Keywords:** Mass spectrometry imaging, Lipidomics, Bioinformatics software, Data processing

## Abstract

**Introduction:**

Mass spectrometry imaging (MSI) experiments result in complex multi-dimensional datasets, which require specialist data analysis tools.

**Objectives:**

We have developed *massPix—*an R package for analysing and interpreting data from MSI of lipids in tissue.

**Methods:**

*massPix* produces single ion images, performs multivariate statistics and provides putative lipid annotations based on accurate mass matching against generated lipid libraries.

**Results:**

Classification of tissue regions with high spectral similarly can be carried out by principal components analysis (PCA) or *k*-means clustering.

**Conclusion:**

*massPix* is an open-source tool for the analysis and statistical interpretation of MSI data, and is particularly useful for lipidomics applications.

## Introduction

Mass spectrometry imaging (MSI) is a transformative technology in systems biology and clinical research (Addie et al. 2015; Angel and Caprioli 2013). MSI enables the in situ analysis of tissue molecular composition for hundreds of metabolites and lipids simultaneously. Sophisticated approaches and software are therefore required in order to analyse and interpret the vast amount of data collected with each imaging experiment. As such, new bioinformatics tools and resources are needed to recreate molecular maps across tissue and probe statistical differences across a tissue slice using advanced pattern recognition tools, particularly in studies where disease processes need to be examined on a spatial basis (Alexandrov et al. 2010; Smentkowski et al. 2007; Van de Plas et al. 2007).

There have been various software packages released to view and analyse MSI data (Bemis et al. 2015; Gibb and Strimmer 2012; Källback et al. 2016; Parry et al. 2013; Verbeeck et al. 2014). Many tools including Biomap, DataCube Explorer, msIQuant and MSiReader do not perform multivariate statistical analysis, whilst others are vendor specific, e.g., ImageQuest (Thermo Scientific). Omnispect and Cardinal are freely available and perform multivariate analysis on data using non-negative matrix factorization and spatially-aware clustering approaches, respectively. However these software packages do not provide lipid feature annotation. Recently, a framework for false-discovery rate-controlled metabolite annotation for MSI has been developed as part of the METASPACE consortium, with great potential for stream-lining MSI data analysis (Palmer et al. 2017).

Here, we have developed *massPix*, an R-based package which processes MSI data, plots single ion distributions and performs multivariate statistics [principal components analysis (PCA) and clustering]. This software is different from available tools, in that it has been designed specifically for lipidomics applications, enabling putative lipid annotations based on accurate mass. In addition, PCA and clustering may be performed to classify regions across tissue based on their lipid profiles (Hall et al. 2016, 2017). Furthermore the software is freely available, easy to implement by novices to R, and adaptable if required, by advanced users.

## Implementation

*massPix* supports data in imzML format (Race et al. 2012; Schramm et al. 2012). Free converters for raw data to imzML are available from http://www.imzML.org. Whilst *massPix* has been developed for high resolution matrix assisted laser desorption ionisation (MALDI) data acquired with Thermo Scientific instrumentation, the software is vendor agnostic and can be applied to any data in imzML format independent of mass spectrometry platform. *massPix* is compatible with Windows, Mac and Linux operating systems, and requires at least sufficient RAM to load the entire experimental dataset into memory (for instance to process 3 GB image file, ~3.2 GB memory is used). *massPix* is run from the R scripting interface, however a detailed knowledge of R is not required to install and use the software. Those with advanced knowledge of R programming can adapt the source code for their own needs. *massPix* outputs high quality images, a data frame of the final normalised and annotated image which can be further manipulated in R, and csv files for spectra corresponding to cluster centers, PCA loadings, and lipid annotations. The *massPix* R package, all R scripts, library files and the imzML Converter are available on GitHub (https://github.com/hallz/massPix). A brief introduction is provided with parameter descriptions, in addition to a step-by-step presentation on software use and instructions on file conversion. Test data is available on the MetaboLights data repository (study ID: MTBLS487).

## Results and discussion

### Data acquisition

**Fig. 1:**
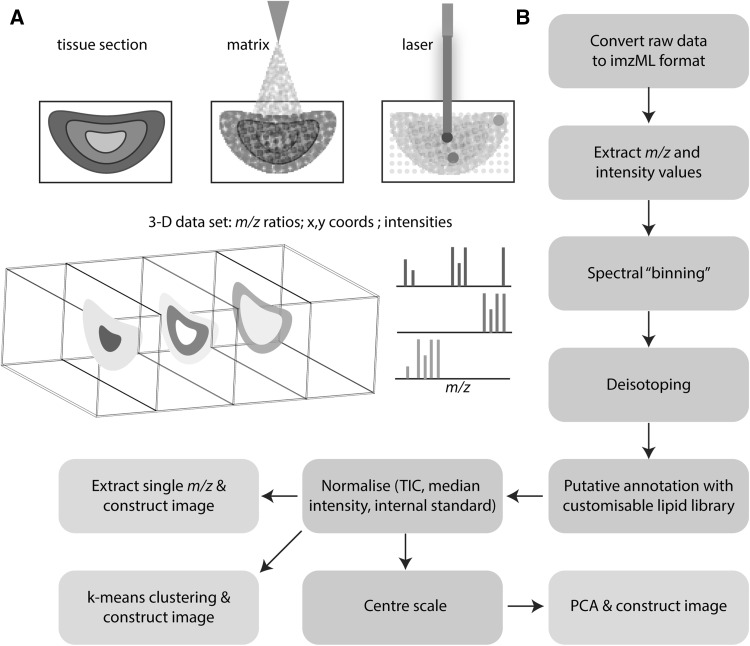
Overall MSI data acquisition and data analysis workflow. One of the most common mass spectrometry imaging approaches uses matrix assisted laser desorption ionisation (MALDI). First, a tissue section is coated with a matrix to aid ionisation. Then a laser is fired across the tissue, generating a spectrum (*m*/*z* ratios, ion intensities) for each pixel analysed (*x, y* coordinate) (**a**). The overall data processing workflow followed by *massPix* is shown (**b**)

Most MSI workflows are based on MALDI or desorption electrospray ionisation (DESI) datasets. MALDI–MSI is currently more widely used within the field and these datasets have been used to developed *massPix*. In MALDI, a matrix is first applied to the tissue surface to aid ionisation. This is typically a small organic molecule, capable of absorbing the wavelength supplied by the laser and subsequently ionising surrounding analyte molecules (Fig. 1a). The laser raster-scans across the tissue surface, generating a mass spectrum for every pixel sampled. Spatial resolution is dependent on the optical design of the instrument, and varies from one to several hundred microns. The datasets generated are multi-dimensional, large and information-rich.

### *massPix* pipeline

The overall data processing workflow (Fig. 1b) consists of initial data pre-processing, filtering, image subsetting, deisotoping, annotation, normalisation, scaling, image “slicing” and multivariate statistics. First raw data must be converted to imzML format, which is then parsed to R. Ions with intensities greater than a threshold, from each spectra, are extracted and grouped to user-adjustable mass bins. The choice of bin width is dependent on the instrument mass resolving power (e.g. 10 ppm bin width for data acquired with 60,000 mass resolution at *m*/*z* 400; for lower/higher resolving power increase or decrease bin width, respectively). Spectral features are defined by the median *m*/*z* value in each bin, and only features detected above a threshold proportion of spectra are retained. Average intensities for all features from a random subset of pixels are computed and used to perform deisotoping. The deisotoping algorithm identifies the molecular ion (M) and removes isotopes at *m*/*z* (M+1) and (M+2) which are within a calculated proportion of the intensity of M.

Putative lipid annotation by accurate mass is achieved by searching deisotoped ions against a generated library of lipid *m*/*z* ratios computed for all combinations of common fatty acids, lipid head-groups and anticipated adducts in each ionisation mode. The criteria for a match can be adjusted according to different MS performance capabilities (for example. <3, <10 ppm etc). Lipid classes searched in positive ion mode are diacylglycerides (DAG), triacylglycerides (TAG), phosphatidylcholines (PC), phosphatidylethanolamines (PE), phosphatidylserines (PS), LysoPC, cholesteryl esters (CE), sphingomyelins (SM) and ceramides (Cer). In negative ion mode, lipid classes searched are PC, phosphatidic acid (PA), PE, PS, phosphatidylglycerols (PG), phosphatidylinositols (PI), and free fatty acids (FFA). Whilst this list is not exhaustive, it does cover the most common lipid classes. Possible adducts considered are [M+K]^+^, [M+H]^+^_,_ [M+Na]^+^, [M+NH_4_]^+^ in positive ion mode and [M–H]^−^, [M+Cl]^−^, [M+OAc]^−^ in negative ion mode. It is important to point out that a database hit based on accurate mass should only be considered the first step in metabolite identification, and confirmation carried out using MS/MS is required, where this appropriate. This is particularly critical where data has been collected at lower mass accuracy, for instance using lower resolution time-of-flight instruments, where the risk of false positives is higher. For example, using the test data provided, an additional 200 possible lipid annotations were made by changing the mass accuracy for annotation from 5 to 50 ppm.

*massPix* has the further capability to perform difference matching on deisotoped features to search for mass differences associated with measurement-introduced alternation (e.g. fragmentation) or biological modifications (e.g. oxidation). Ion intensities are then normalised either to the median or total ion count, or to the average intensity of a set of standard ions. Single ion images can be produced, or normalised intensities used to create multivariate statistical images based on *k*-means clustering or PCA following centering and Pareto scaling (van den Berg et al. 2006). The analysis can be readily customised by replacing default parameters for filtering, normalisation and scaling, library composition, lipid assignment and image reporting.

### Test data

**Fig. 2:**
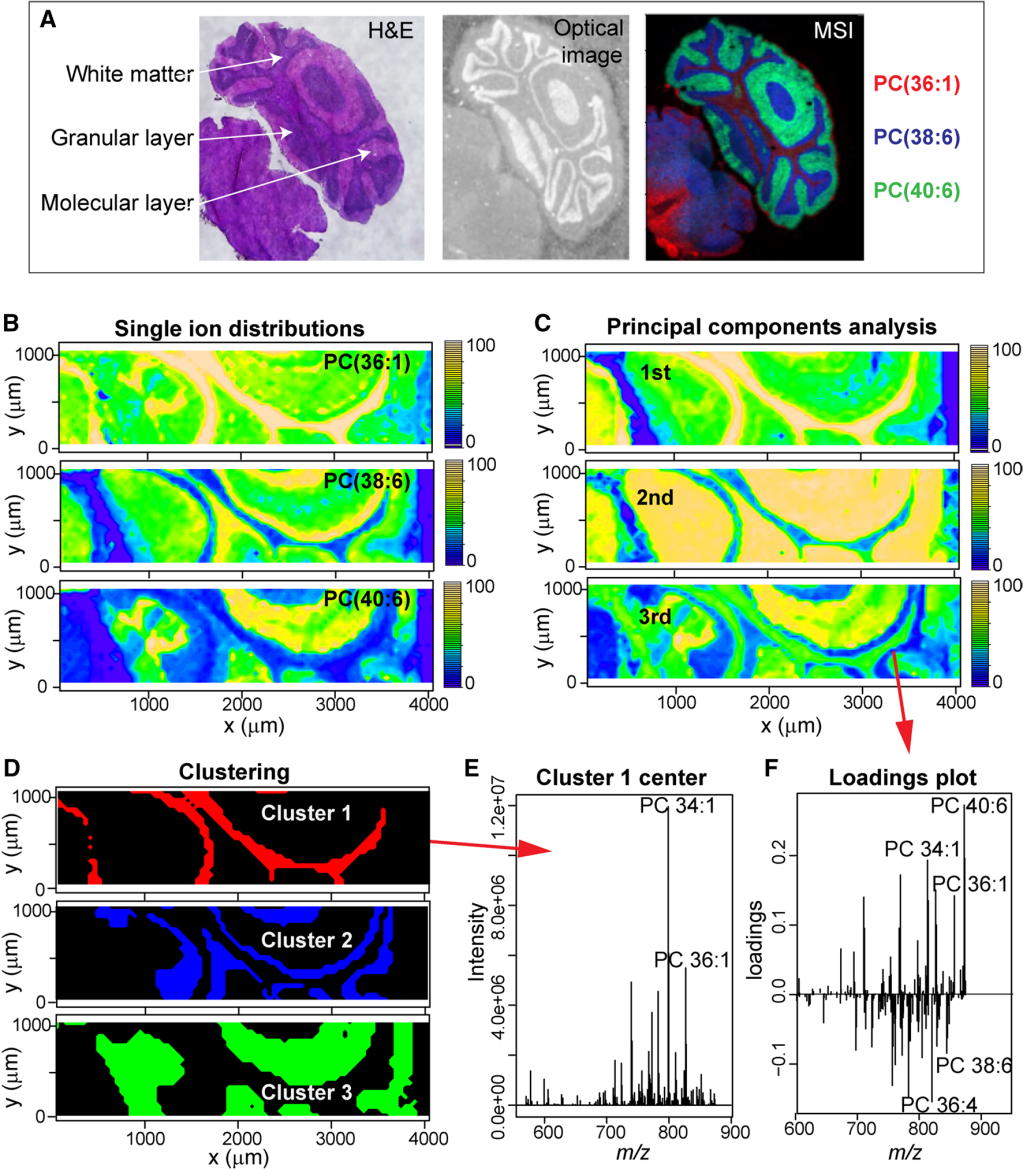
Imaging mouse cerebellum using MALDI-MSI. H&E stained section of mouse cerebellum, with major tissue regions highlighted (*left*). An adjacent section was coated in matrix (*middle*) and analysed by MSI (*right*). Single ion distributions for [PC(36:1)+K]^+^, [PC(38:6)+K]^+^_,_ [PC(40:6)+K]^+^, shown in *red, blue* and *green*, are predominantly located in white matter, granular layer and molecular layer, respectively (**a**). Overlaid image produced using ImageQuest (Thermo Scientific). Single ion distributions produced by *massPix* for [PC(36:1)+K]^+^, [PC(38:6)+K]^+^_,_ [PC(40:6)+K]^+^, in a sub-section of cerebellum (**b**). Principal components analysis (PCA) (**c**) and *k*-means clustering (**d**) differentiate regions based on their lipid profiles. Average spectra for pixels located in cluster 1 (**e**). PCA loadings plot for the third principal component (**f**); lipids with more positive (negative) loadings correspond to regions with higher (lower) principal component scores

The power of multivariate statistics allows the differentiation of regions within tissue based on their lipid composition. This allows one to compare different regions in the same slice of tissue, for example tumour and adjacent tissue. As a test dataset, 15 micron tissue sections of wild type mouse cerebellum were coated with 2,5-dihydroxybenzoic acid (DHB) matrix (Sigma Aldrich, St Louis, MO; 10 mg/mL) and analysed by MSI (MALDI LTQ Orbitrap XL, Thermo Scientific, Hemel Hempstead, UK). The three major tissue regions within the cerebellum - white matter, granular and molecular layers (Fig. 2a)—were clearly differentiated by specific lipid profiles. Single ion distributions are shown for [PC(36:1)+K]^+^ (MSI Level 2; ChEBI:66857), [PC(38:6)+K]^+^ (MSI Level 2; ChEBI:64519), [PC(40:6)+K]^+^ (MSI Level 2; ChEBI:64431) which are predominantly located in white matter, granular layer and molecular layers, respectively (Fig. 2a, b). *massPix* uses an unsupervised approach to classify pixels of high spectral similarity using PCA (Fig. 2c) and *k*-means clustering (Fig. 2d). Spectra of cluster centres (Fig. 2e) and PCA loadings plots (Fig. 2f) provide detailed information about the relative lipid profiles of distinct regions and which lipid species are important for classification. The use of *massPix* software can thus aid interpretation of region-specific molecular changes. This is particularly important for understanding molecular mechanisms in disease processes.
